# Radiotherapy and Anrotinib in Malignant Glomus Tumor of the Bladder: A Case Report and Literature Review

**DOI:** 10.1089/cbr.2023.0151

**Published:** 2024-04-24

**Authors:** Jing Ai, Shuang Zhang, Yimeng Qian, Lin Kang, Litao Zhang, Jing Zhao

**Affiliations:** ^1^ Department of Oncology, Hebei General Hospital, Shijiazhuang, China.; ^2^ Graduate School, North China University of Science and Technology, Tangshan, China.; ^3^ Graduate School, Hebei North University, Zhangjiakou, China.; ^4^ Department of Pathology, and Hebei General Hospital, Shijiazhuang, China.; ^5^ Department of Emergency, Hebei General Hospital, Shijiazhuang, China.

**Keywords:** malignant glomus tumors, radiation therapy, iodine-125 seed implantation, transcatheter arterial chemoembolization, antiangiogenic therapy

## Abstract

**Background:**

Malignant glomus tumors (MGTs) are rare malignancies, which grow rapidly and are aggressive. Surgical resection has been regarded as the standard management, but treatment options for those unresectable tumors are limited, resulting in a high recurrence rate and poor prognosis.

**Case Description:**

An 85-year-old man presented with gross hematuria and was diagnosed with MGTs of bladder. The patient achieved long-term local control after multimodal therapy comprising radiotherapy, iodine-125 seeds brachytherapy, transcatheter arterial chemoembolization, and antiangiogenic targeted therapy.

**Conclusion:**

MGTs occurring in the bladder are clinically rare and refractory. The case presented here highlights the importance of multidisciplinary diagnosis and treatment, providing evidence that radiotherapy and antiangiogenic therapy may play an important role in unresectable bladder MGT.

## Introduction

Bladder malignancy is the 10th most common malignant tumor in the world, with >90% being urothelial carcinoma, followed by squamous cell carcinoma and adenocarcinoma. Glomus tumor (GT) is a mesenchymal neoplasm composed of cells resembling the perivascular modified smooth muscle cells of the normal glomus body.^1^ GTs account for <2% of soft tissue tumors, most are benign and <1% are malignant.^2^ Malignant GT (MGT) occurring in the bladder is extremely rare, and only one case of bladder MGT was reported previously.^3^ The traditional treatment for bladder carcinoma includes surgery, radiotherapy, and chemotherapy.

Immunotherapy represented by immune checkpoint inhibitors and drugs of antibody–drug conjugate has developed rapidly in recent years and is considered new treatment options.^4,5^ However, the treatments of inoperable MGTs have not been reported. In this study, a patient was reported with unresectable bladder MGT who has achieved long-term local control through multimodal treatment including radiotherapy, iodine-125 brachytherapy, transcatheter arterial chemoembolization (TACE), and antiangiogenic systemic treatment, to enhance understanding of MGTs and improve treatment strategies for bladder MGT.

## Case Presentation

**FIG. 1.**
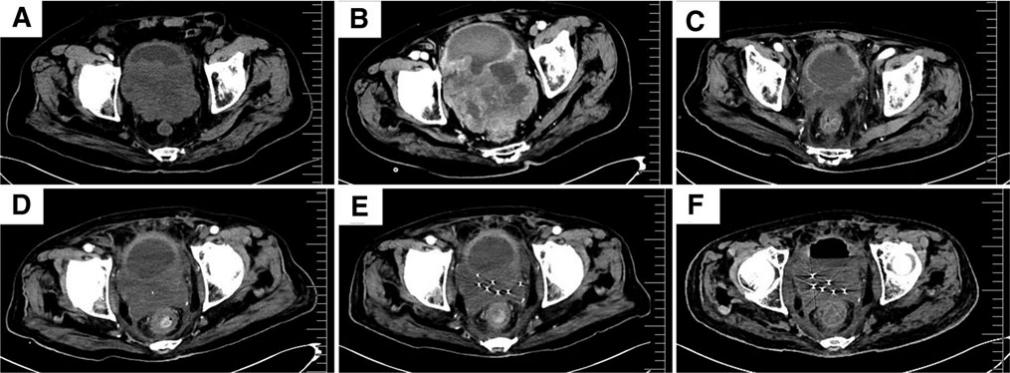
Computed tomography. **(A)** Obvious occupying lesion in the posterior bladder wall. **(B)** Obvious enlargement of the occupying lesion in the postoperative bladder wall. **(C)** Obvious reduction of the lesion seen after radiotherapy. **(D)** Disease progression 3 months after radiotherapy. **(E)** After particle implantation. **(F)** No progression after antiangiogenic therapy.

An 85-year-old man was admitted in December 2021 due to gross hematuria, and physical examination showed no special signs. Computed tomography (CT) scan revealed a mass on the posterior wall of bladder with nonhomogeneous enhancement and the boundary between the posterior wall of the bladder and the prostate was unclear (Fig. 1A). After multidisciplinary treatment discussion, considering the patient's advanced age, poor physical condition, and prostate involvement, the patient had no indication for radical resection and received partial tumor resection and biopsy under cystoscopy on January 10, 2022.

**FIG. 2.**
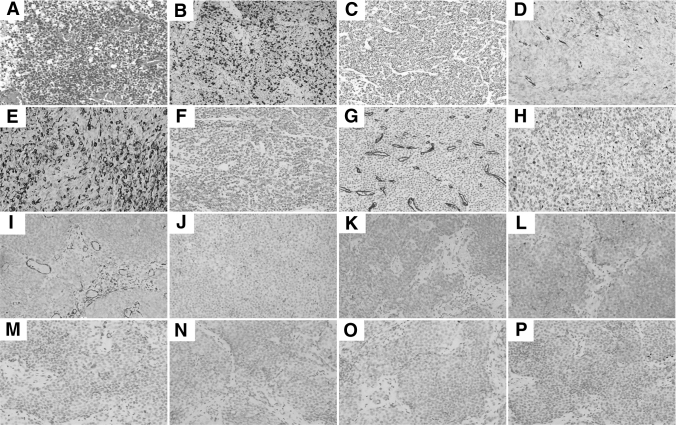
The HE and immunohistochemical pictures: **(A)** (HE, original magnification, 100 × ). **(B)** Ki-67 (40–60%+, 100 × ). **(C)** SMA (+, 100 × ). **(D)** Calponin (+, 100 × ). **(E)** H-caldesmon (+, 100 × ). **(F)** CKpan (−, 100 × ). **(G)** CD34 (−, 100 × ). **(H)** Desmin (−, 100 × ). **(I)** Vimentin (−, 100 × ). **(J)** S100 (−, 100 × ). **(K)** EMA (−, 100 × ). **(L)** GATA (−, 100 × ). **(M)** CK7 (−, 100 × ). **(N)** CK20 (−, 100 × ). **(O)** P40 (−, 100 × ). **(P)** P63 (−, 100 × ). EMA, epithelial membrane antigen; HE, hematoxylin–eosin; SMA, smooth muscle antigen.

Pathological hematoxylin–eosin staining showed a small round cell malignant tumor with multifocal necrosis. Immunohistochemistry revealed the following (Fig. 2): Ki-67(40–60%+), smooth muscle antigen (SMA)(+), calponin(+), H-calponin(+), CKpan(−), CD34(−), desmin(−), vimentin(−), S100(−), epithelial membrane antigen(−), GATA binding factor 3(−), CK7(−), CK20(−), P40(−), P63(−), CD56(−), CK34(−), CK5/6(−), Syn(partial+), CgA(−), PSA(−), SSTR2(−), ATPX(+), CD117(−), and CD38(−), which supported the diagnosis of MGTs. In March 2022, CT re-examination showed the tumor was larger than before (Fig. 1B).

The patient refused systemic chemotherapy. Then, volumetric-modulated arc therapy was administered with a linear accelerator using 6-MV photons to deliver a total dose of 60 Gy in 30 fractions over 6 weeks from March 25, 2022, to May 11, 2022. A CT scan performed 1 month after radiotherapy showed that the tumor was significantly reduced and reached partial remission (Fig. 1C). Four months after radiotherapy, in September 2022, a follow-up CT scan showed that the tumor increased compared with that in June 2022 (Fig. 1D). Subsequently, the patient underwent CT and ultrasound-guided radioactive iodine-125 seeds implantation, and 50 seeds with an activity of 0.7 mCi were implanted.

The matched peripheral dose was 65.54 Gy (Fig. 1E). Furthermore, genetic testing was performed on the tumor tissue, and the results showed mutations in *TP53*, *VEGFA*, and *VEGFR2*. Then, targeted antiangiogenic therapy was initiated with oral anlotinib 8 mg per day for 14 days, every 21 days. More than 1 month later, the patient intermittently developed bloody stools, and stopped using anlotinib. Colonoscopy examination revealed a new mass ∼2 cm from the anal margin, and pathological examination confirmed it still to be MGT.

Immunohistochemistry showed CKpan (−), vimentin (−), SMA (+), Syn (−), CDX-2 (−), CgA (−), and CEA (−). Owing to bloody stools, the patient underwent TACE in October 2022, during which 40 mg of pirarubicin was infused into the supplying artery. After TACE treatment, bloody stool disappeared, so anlotinib was continued. A CT scan conducted in December 2022 showed that the tumor size remained stable without significant changes compared with that in September 2022 (Fig. 1F). Unfortunately, 1 month later in January 2023, the patient died of COVID-19 pneumonia. The overall survival time from diagnosis of MGTs to death was 12 months.

## Discussion

GTs are rare mesenchymal neoplasms composed of cells resembling the perivascular modified smooth muscle cells of the normal glomus body,^1^ most of which occur in the dermis and rarely in the visceral organs.^6^ GTs account for <2% of soft tissue tumors, most are benign and <1% are malignant.^2^ Only five cases of bladder GTs have been reported so far, and only one of them is malignant (Table 1).^3,7–10^ The clinical presentation of GTs varies according to the site of occurrence.

**Table 1.** Clinicopathological Data of Reported Glomus Tumor Cases of Bladder

Author (year)	Age	Sex	Tumor nature	Main symptoms	Metastasis site	Tumor site	Size	Treatment	Prognosis
Tripodi et al. (2013)	63	M	Benign	Gross hematuria	No	Anterior wall of bladder	Maximum diameter 1.2 cm	TURBT	Follow-up for 1 year showed no progress.
Chalise et al. (2012)	44	M	Glomus tumor of uncertain malignant potential	Gross hematuria	No	bladder	1.6 × 1.3 × 0.7 cm	TURBT	No mention in the text.
Chen et al. (2021)	57	F	Benign	Gross hematuria	No	Right side wall of bladder	Maximum diameter 0.6 cm	TURBT	Follow-up for 2 years showed no progress.
Palmisano et al. (2017)	58	M	Symplastic glomus tumor	An asymptomatic mass	No	Anterior wall of bladder	2.5 × 2.5 cm	Robot-assisted partial cystectomy	Follow-up for 1 years showed no progress.
Shim et al. (2005)	57	F	Malignant	Gross hematuria	Multiple pulmonary metastases	Left side wall of bladder	6.5 × 5 × 5.8 cm	TURBT and chemotherapy	Died 2 months after diagnosis

F, female; M, male; TURBT, transurethral resection of bladder tumor.

GTs occurring in the bladder are characterized by gross hematuria. Pathological diagnosis is the gold standard. Immunohistochemistry of GTs shows expression of smooth muscle actin and/or panmuscle actin, CD34, h-caldesmon, and type IV collagen.^11^ In 2001, Flope et al. proposed the diagnostic criteria of MGTs: tumors with a deep location and a size of >2 cm, or atypical mitotic figures, or moderate-to-high nuclear grade and 5 mitotic figures/50 high power field.^11^ The patient met the diagnostic criteria for MGTs.

Owing to the rarity of GTs, there are no guideline recommendations or expert consensus on its treatment principles. Surgical excision is recommended for benign GTs. For MGTs, systemic treatment such as surgery, radiotherapy, and chemotherapy can be considered. The case presented is an unresectable bladder MGT, he received 60 Gy dose of radiotherapy with significantly reduced tumor size, and a progression-free survival (PFS) of 4 months (Fig. 1C).

However, the tumor progressed and invaded the anterior wall of the rectum 4 months after the end of radiotherapy. Therefore, radiotherapy is considered an effective treatment, but the dose of 60 Gy is insufficient for bladder MGTs and a higher dose is required for local radical treatment. When a high radical dose cannot be implemented due to dose limitations on organs at risk (OARs) around the tumor, radioactive iodine-125 seeds can be applied for local radiation dose boost.

Chemotherapy regimens for MGTs tend to be similar to those used for sarcomas.^12^ This patient presented with rectal invasion resulting in hematochezia, which was alleviated after TACE treatment with pirarubicin and tumor was stable. For targeted therapy, the current research data are very limited, a previous study suggested that *B-Raf proto-oncogene* mutations may be present in MGTs.^13^ Refer to the genetic test results of this patient, which show *VEGFA* and *VEGFR2* mutations, the patient received antiangiogenic targeted therapy with anlotinib and the PFS reached 4 months. Antiangiogenic targeted therapy is considered effective for MGTs.

## Conclusion

MGTs occurring in the bladder is clinically rare and refractory, with a high recurrence rate and poor prognosis. This case provides evidence that radiotherapy may be an alternative to surgery for bladder MGTs, especially for those that cannot be radically resected. The prescribed dose of radiotherapy can refer to soft tissue sarcomas, which needs to exceed 60 Gy. When the target dose is insufficient due to OARs, Iodine-125 seeds brachytherapy can be supplemented. Moreover, antiangiogenic targeted therapy would be a promising therapy for MGTs. However, further studies are still needed.

## References

[1] Gombos Z, Zhang PJ. Glomus tumor. Arch Pathol Lab Med 2008;132(9):1448–1452; doi: 10.5858/2008-132-1448-GT 18788860

[2] Lamba G, Rafiyath SM, Kaur H, et al. Malignant glomus tumor of kidney: The first reported case and review of literature. Hum Pathol 2011;42(8):1200–1203; doi: 10.1016/j.humpath.2010.11.009 21333326

[3] Shim HS, Choi YD, Cho NH. Malignant glomus tumor of the urinary bladder. Arch Pathol Lab Med 2005;129(7):940–942; doi: 10.5858/2005-129-940-mgtotu 15974822

[4] Kim JH, Chang IH. A novel strategy for treatment of bladder cancer: Antibody-drug conjugates. Investig Clin Urol 2022;63(4):373–384; doi: 10.4111/icu.20220061 PMC9262489 35670004

[5] Rizzo A, Mollica V, Massari F. Expression of programmed cell death ligand 1 as a predictive biomarker in metastatic urothelial carcinoma patients treated with first-line immune checkpoint inhibitors versus chemotherapy: A systematic review and meta-analysis. Eur Urol Focus 2022;8(1):152–159; doi: 10.1016/j.euf.2021.01.003 33516645

[6] Wan PZ, Han Q, Wang EH, et al. Glomus tumor of uncertain malignant potential of the lung: A case report and review of literature. Int J Clin Exp Pathol 2015;8(11):15402–15406. 26823902 PMC4713688

[7] Chalise S, Jha A, Neupane PR. Glomangiomyoma of uncertain malignant potential in the urinary bladder: A case report. JNMA J Nepal Med Assoc 2021;59(239):719–722; doi: 10.31729/jnma.5388 34508517 PMC9107852

[8] Chen L, Lai B, Su X, et al. Unusual glomus tumor of the bladder: A rare case report and literature review. BMC Urol 2021;21(1):66; doi: 10.1186/s12894-021-00837-0 33882895 PMC8061168

[9] Palmisano F, Gadda F, Spinelli MG, et al. Symplastic glomus tumor of the urinary bladder treated by robot-assisted partial cystectomy: A case report and literature review. Urologia 2018;85(3):130–132; doi: 10.5301/uro.5000214 28106242

[10] Tripodi SA, Rocca BJ, Mourmouras V, et al. Benign glomus tumor of the urinary bladder. Arch Pathol Lab Med 2013;137(7):1005–1008; doi: 10.5858/arpa.2012-0125-CR 23808474

[11] Folpe AL, Fanburg-Smith JC, Miettinen M, et al. Atypical and malignant glomus tumors: Analysis of 52 cases, with a proposal for the reclassification of glomus tumors. Am J Surg Pathol 2001;25(1):1–12; doi: 10.1097/00000478-200101000-00001 11145243

[12] Wolter NE, Adil E, Irace AL, et al. Malignant glomus tumors of the head and neck in children and adults: Evaluation and management. Laryngoscope 2017;127(12):2873–2882; doi: 10.1002/lary.26550 28294349

[13] Karamzadeh Dashti N, Bahrami A, Lee SJ, et al. BRAF V600E mutations occur in a subset of glomus tumors, and are associated with malignant histologic characteristics. Am J Surg Pathol 2017;41(11):1532–1541; doi: 10.1097/PAS.0000000000000913 28834810

